# Combined crystal-storing histiocytosis, light chain proximal tubulopathy, and light chain crystalline podocytopathy in a patient with multiple myeloma: a case report and literature review

**DOI:** 10.1080/0886022X.2022.2145970

**Published:** 2023-01-12

**Authors:** Li Zhu, Lei Wang, Hongxia Shi, Lei Jiang, Xin Li, Chunying Shao, Yu Yan, Bao Dong, Wanzhong Zou, Li Zuo

**Affiliations:** a Department of Nephrology, Peking University People’s Hospital, Beijing, China; b Electron Microscope Lab, Peking University People’s Hospital, Beijing, China; c National Clinical Research Center for Hematologic Disease, Peking University People’s Hospital, Peking University Institute of Hematology, Beijing, China

**Keywords:** Crystal-storing histiocytosis, multiple myeloma, light chain proximal tubulopathy, light chain crystalline podocytopathy, monoclonal immunoglobulin-induced crystalline nephrology

## Abstract

**Background:**

Crystal-storing histiocytosis (CSH), light chain proximal tubulopathy (LCPT), and light chain crystalline podocytopathy (LCCP) are rare complications of multiple myeloma (MM) or monoclonal gammopathy of renal significance, and their diagnoses are challenging.

**Case presentation:**

In this case, a 69-year-old Chinese woman presented with suspicious Fanconi syndrome with renal insufficiency. Immunofixation electrophoresis of both serum and urine revealed elevated immunoglobulin G kappa (IgGkappa) and kappa light chain. Bone marrow aspirate revealed 15% plasma cells with considerable cytoplasmic granular inclusions and needle-shaped crystals. Renal biopsy confirmed the final pathologic diagnosis of kappa-restricted CSH, combined LCPT and LCCP by immunoelectron microscopy. A number of special casts were present which could easily be misdiagnosed as light chain cast nephropathy. Immunofluorescence on frozen tissue presented false negative for kappa light chain, as ultimately proven by paraffin-embedded tissue after pronase digestion. MM and CSH were diagnosed, and two cycles of chemotherapy were given. The patient subsequently refused further chemotherapy, and her renal function remained relatively stable during a 2.5-year follow-up period.

**Conclusions:**

In conclusion, we report a rare case of generalized kappa-restricted CSH involving bone marrow and kidney, combined with LCPT and LCCP, provide a comprehensive summary of renal CSH, and propose a new nomenclature of monoclonal immunoglobulin-induced crystalline nephrology. The presentation of monoclonal immunoglobulin and Fanconi syndrome should suggest the presence of monoclonal immunoglobulin-induced crystalline nephrology. Use of paraffin-embedded tissue after pronase digestion and immunoelectron microscopy is beneficial to improve the sensitivity of diagnosis.

## Background

Multiple myeloma (MM) is a hematological malignant tumor that occurs frequently in middle-aged and elderly patients. It accounts for 10% of hematological malignancies and is often accompanied by kidney injury. Renal biopsy is crucial for diagnosis and to evaluate the prognosis of any renal impairment induced by MM [1]. The most common renal impairment in MM is cast nephropathy (CN) [2]. Crystal-storing histiocytosis (CSH) is a rare complication of MM and other B-lymphocyte proliferative diseases, and can involve a wide variety of organs. The most common target is bone marrow, followed by the head and neck, with kidney ranking third [3]. To date, only 28 cases of CSH with renal involvement have been reported in the literature [4–27], some of which presented with other crystalline deposits, such as light chain proximal tubulopathy (LCPT) and light chain crystalline podocytopathy (LCCP). It is difficult to recognize and diagnose this condition because of the very low incidence and the high false-negative rate of crystals in immunofluorescence on frozen tissues [9].

Here, we report a rare case of MM with CSH involving both the bone marrow and kidney, which is complicated by LCPT and LCCP. We also provide a literature review of CSH with renal involvement in order to improve recognition and accuracy of diagnosis. Finally, a new nomenclature of monoclonal immunoglobulin-induced crystalline nephrology is proposed.

## Case presentation

A 69-year-old Chinese woman had intermittent lower back and leg pain for 6 months without obvious causes, and no significant improvement was observed after 1 month of treatment with traditional Chinese medicine. She experienced nausea and vomiting and presented to the local hospital. Laboratory studies revealed hemoglobin levels of 112 g/L (reference, 115–150 g/L), platelet of 90 × 10^9^/L (reference, 125–350 × 10^9^/L), serum creatinine of 11.10 mg/dL (reference, 0.51–0.95 mg/dL), potassium of 3.05 mmol/L (reference, 3.50–5.30 mmol/L), and bicarbonate 10.4 mmol/L (reference, 22.0–29.0 mmol/L). Levels of blood glucose, uric acid, and phosphorus were normal. After hemodialysis, the patient was referred to our hospital. She had no history of chronic disease, and her renal function was normal three years ago.

After admission, the patient’s serum creatinine level pre-dialysis fell to 5.63 mg/dL (normal, 0.51–0.95 mg/dL) without any specific treatment. The serum calcium level was 2.21 mmol/L (reference, 2.20–2.65 mmol/L). A morning urinalysis revealed 2+ protein, 2+ glucose, urine specific gravity of 1.006, and normal blood glucose. Renal glycosuria, with normal serum phosphorus, normal serum uric acid, low serum bicarbonate levels, and continuous hypokalemia under severe renal insufficiency raised clinical suspicion of Fanconi syndrome. The protein level in a 24-h urine sample was elevated to 2.66 g/day (normal, 0.03–0.14 g/day). Urine protein electrophoresis revealed mixed overflow proteinuria, tubular proteinuria, and glomerular proteinuria. Serum protein electrophoresis revealed monoclonal gamma paraprotein (8.4 g/L). Both serum and urine immunofixation electrophoresis revealed restricted immunoglobulin G kappa (IgGkappa) and kappa light chain. Serum free light chain results were κ > 4850 mg/L (normal, 3.30–19.40 mg/L), free λ 61.8 mg/L (normal, 5.71–26.3 mg/L), and κ/λ > 78.48 (normal, 0.26–1.65). Ultrasound showed bilateral renal atrophy (right kidney, 8.7 cm in length; left kidney, 8.8 cm in length). Although the patient did not suffer any trauma, an X-ray of the lumbar spine revealed a pathological fracture of L1, which was not seen three weeks ago. MM was suspected due to the presence of monoclonal gamma paraprotein, a pathological fracture, anemia, and renal impairment. Kidney and bone marrow biopsies were performed.

**Figure 1.**
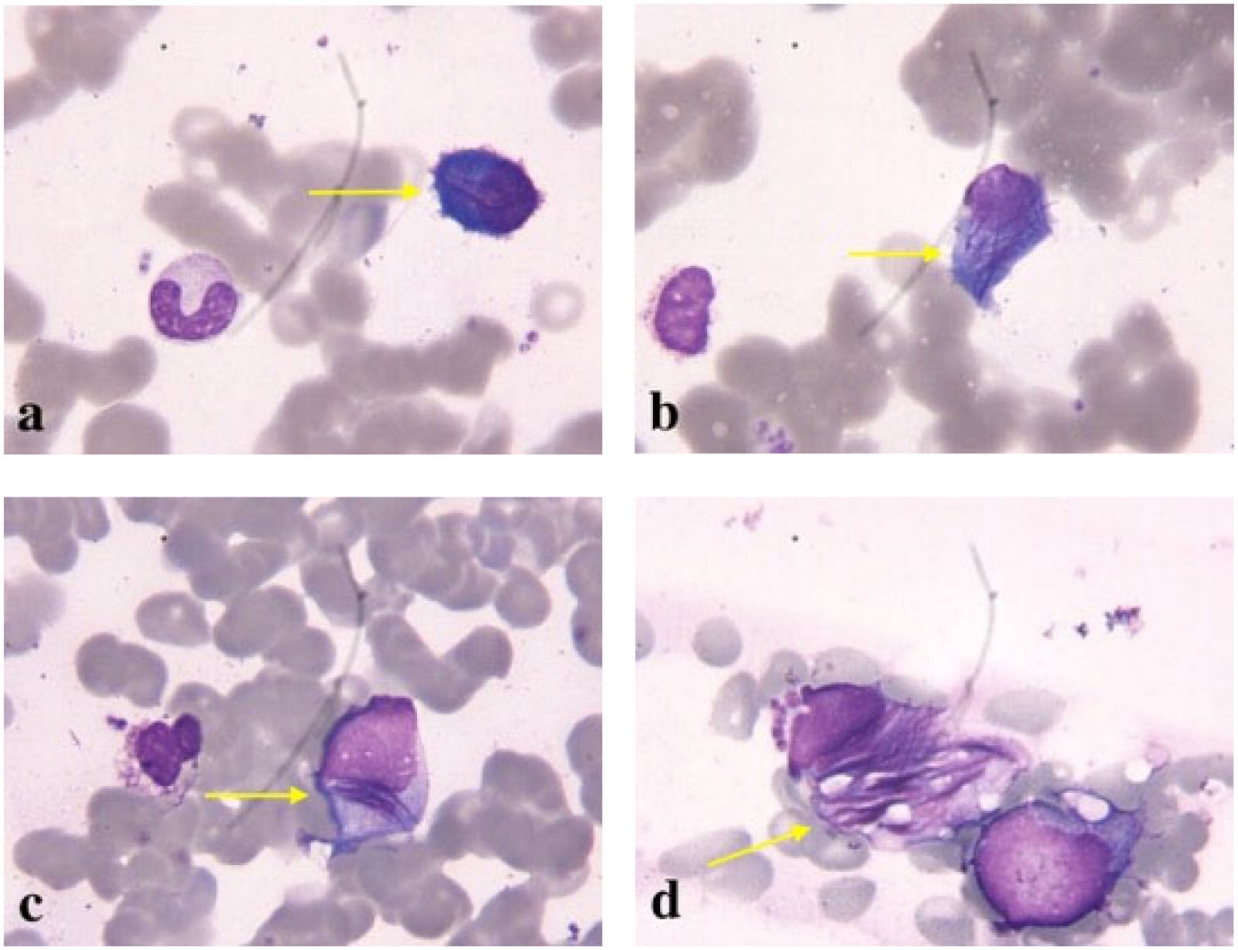
A large number of purple granular inclusions and needle-shaped crystals could be seen in the cytoplasm of bone marrow plasma cells (Wright Giemsa stain, 1000 × original magnification).

Bone marrow aspirate showed plasma cells accounting for approximately 15% (including immature plasma cells account for 5%) of total marrow cellularity, with cytoplasmic granular inclusions and needle-shaped crystals (Figure 1). Flow cytometry analysis performed on a bone marrow aspirate sample identified a monotypic, cytoplasmic kappa light chain-restricted CD38+, CD138+ plasma cell population. Cytogenetic analysis revealed 46, XX. *IGH/CCND1* translocation was confirmed by fluorescence *in situ* hybridization analysis, while *IGH/MAF* and *IGH/FGFR3* translocations were not found. The above findings led to a diagnosis of MM, staged as Durie-Salmon IIB and Revised Multiple Myeloma International Staging System (R-ISS) III [1].

**Figure 2.**
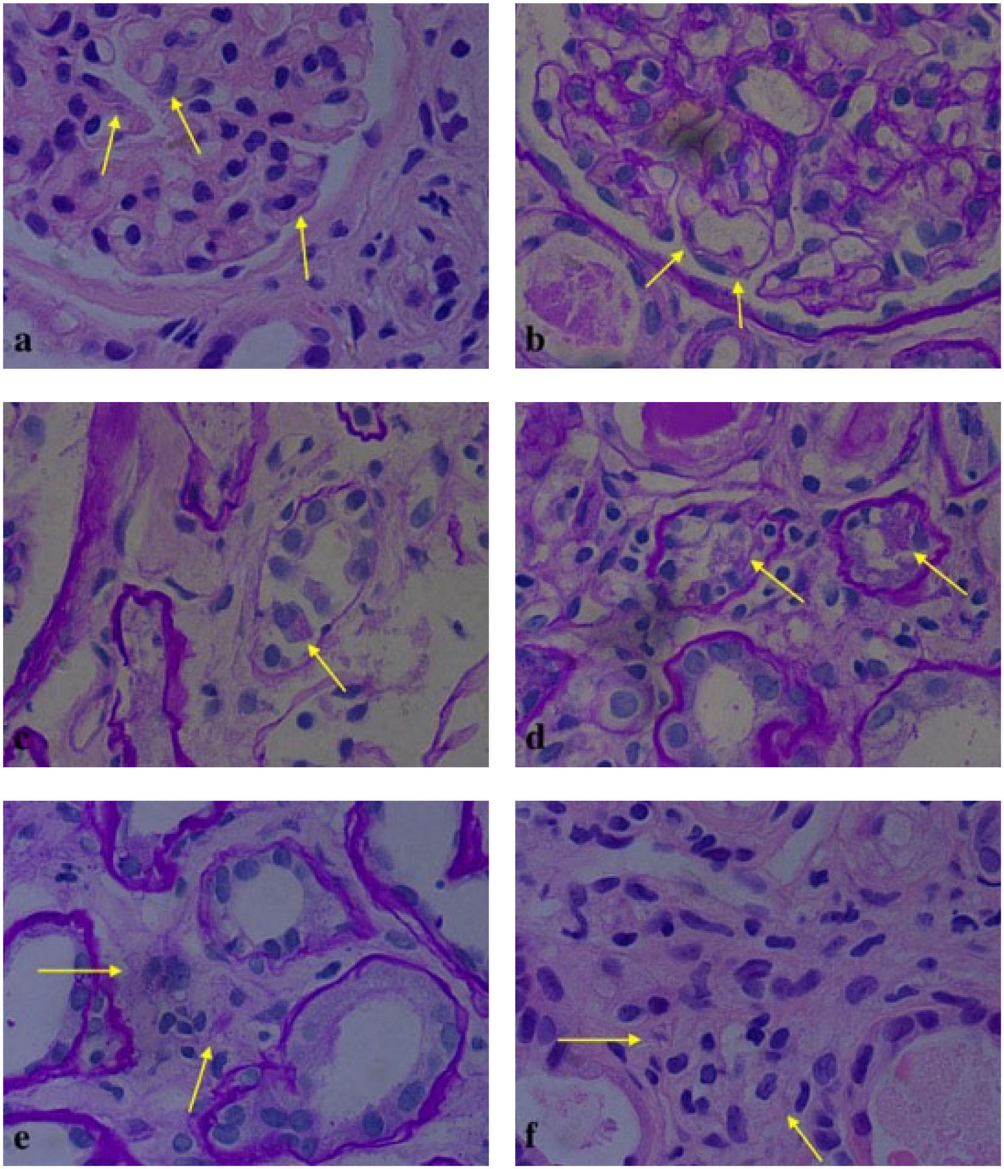
Crystalline inclusions in podocytes (a: HE; b: PAS), proximal tubular epithelial cells (c, d: PAS) and histiocytes (e: PAS; f: HE) (original magnification all ×1000).

**Figure 3.**
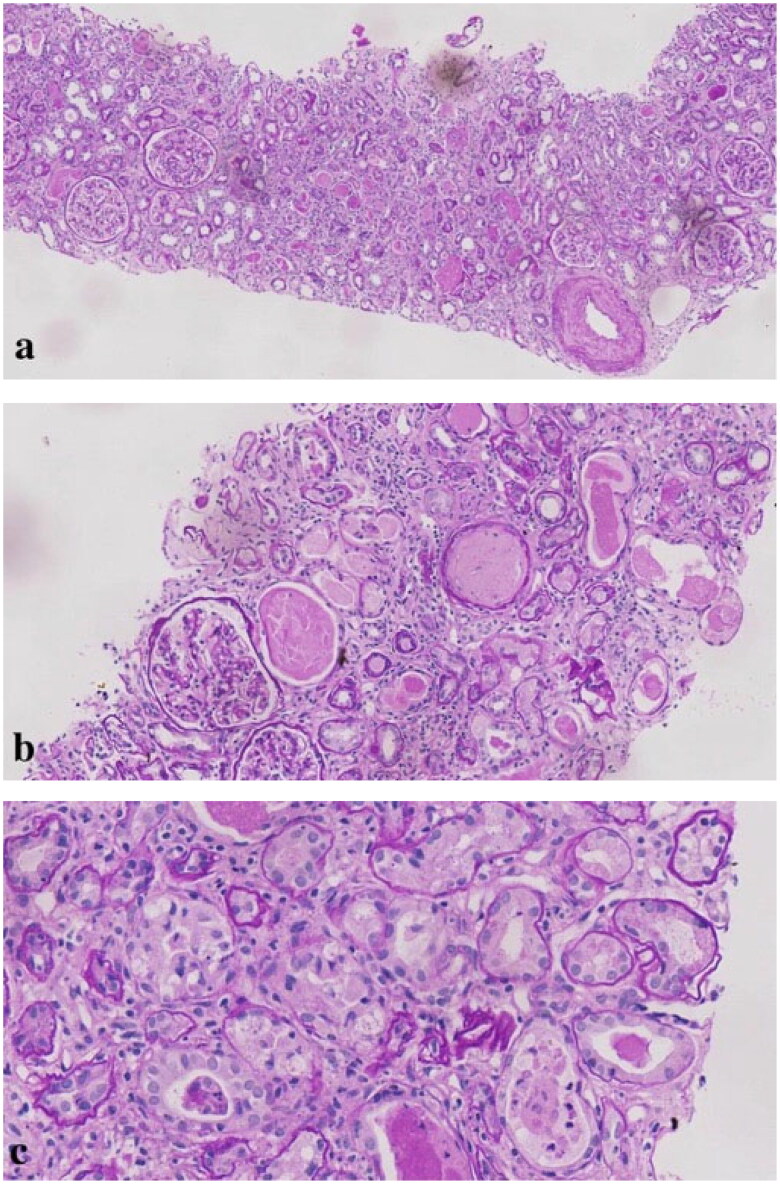
Periodic acid–Schiff (PAS)-positive, heterogeneous, and granular casts could be seen in the tubular lumens (a: PAS, original magnification × 100; b: PAS, original magnification × 200; c: PAS, original magnification × 400).

**Figure 4.**
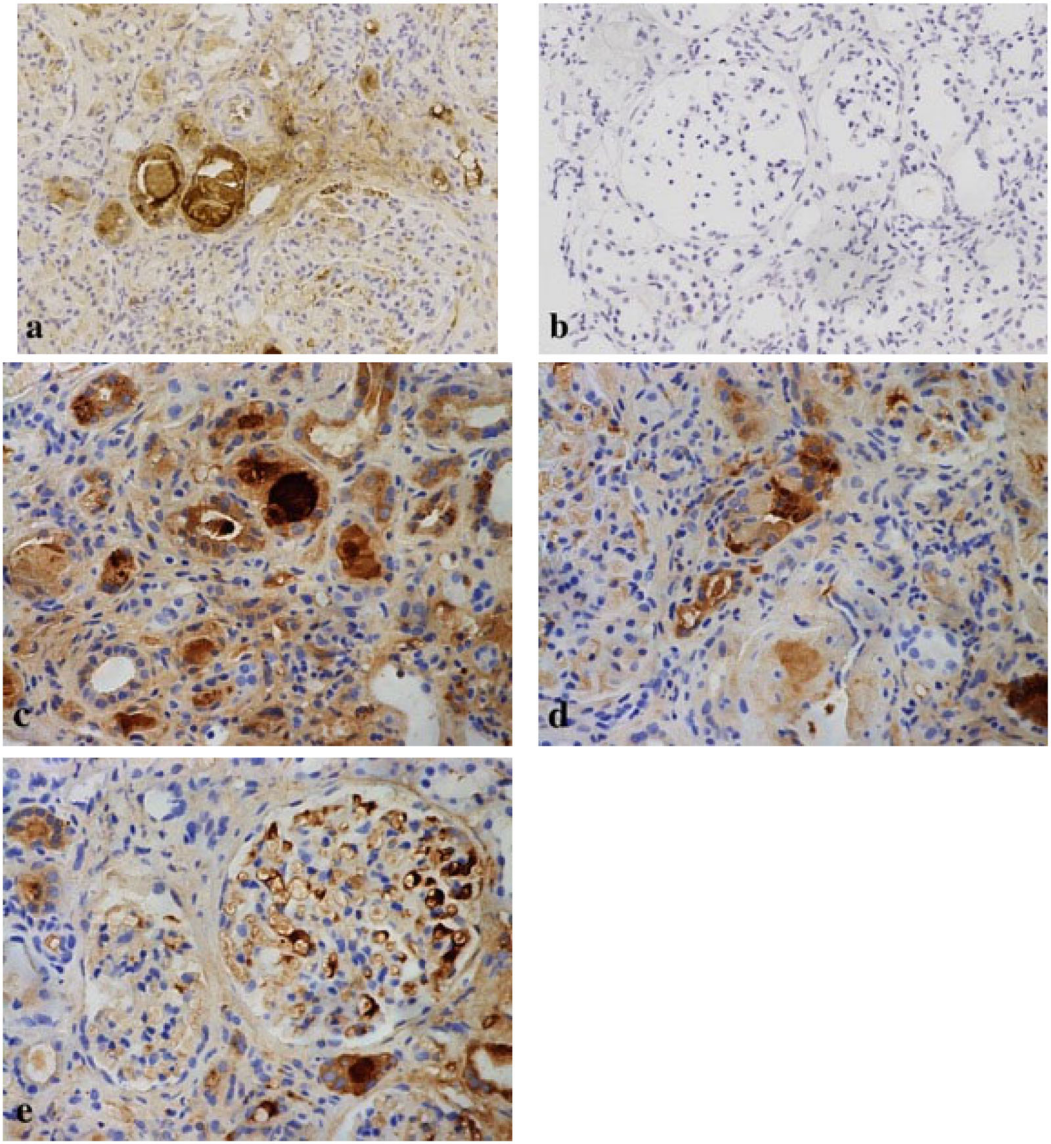
On immunohistochemistry, kappa light chain-restricted expression (a: κ, 200 × original magnification) while lambda was negative (b: λ, 200 × original magnification) in proximal tubular epithelial cells (c: κ, 400 × original magnification), histiocytes (d: κ, 400 × original magnification), and podocytes (e: κ, 400 × original magnification).

**Figure 5.**
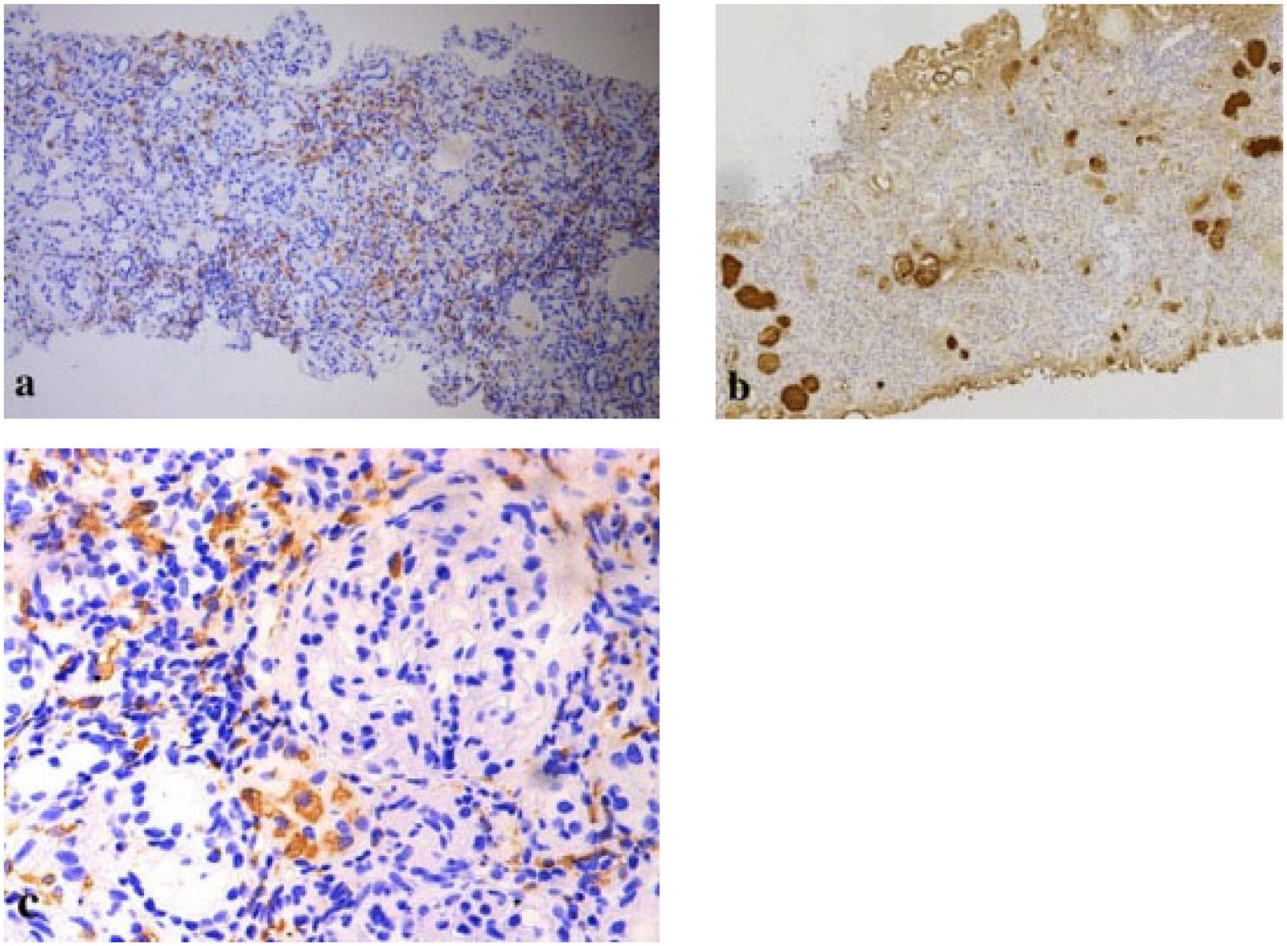
Immunohistochemical stains for immunoglobulins confirm the nature of the crystalline inclusions within the histiocytes. Immunoreactivity for kappa light chain is strong within the histiocytes as well as in the plasma cells, which demonstrates kappa light chain restriction. Immunohistochemistry showed that there were histiocytosis in renal interstitium (a: CD68, 100 × original magnification; b: κ, 100 × original magnification; c: CD68, 400 × original magnification).

**Figure 6.**
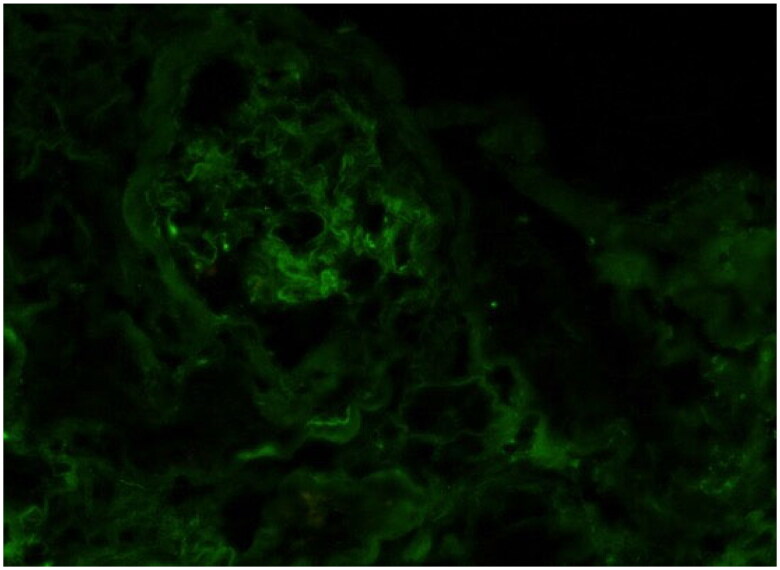
Immunofluorescence with pronase digestion on formalin-fixed paraffin-embedded tissue shows bright staining for kappa (400 × original magnification).

**Figure 7.**
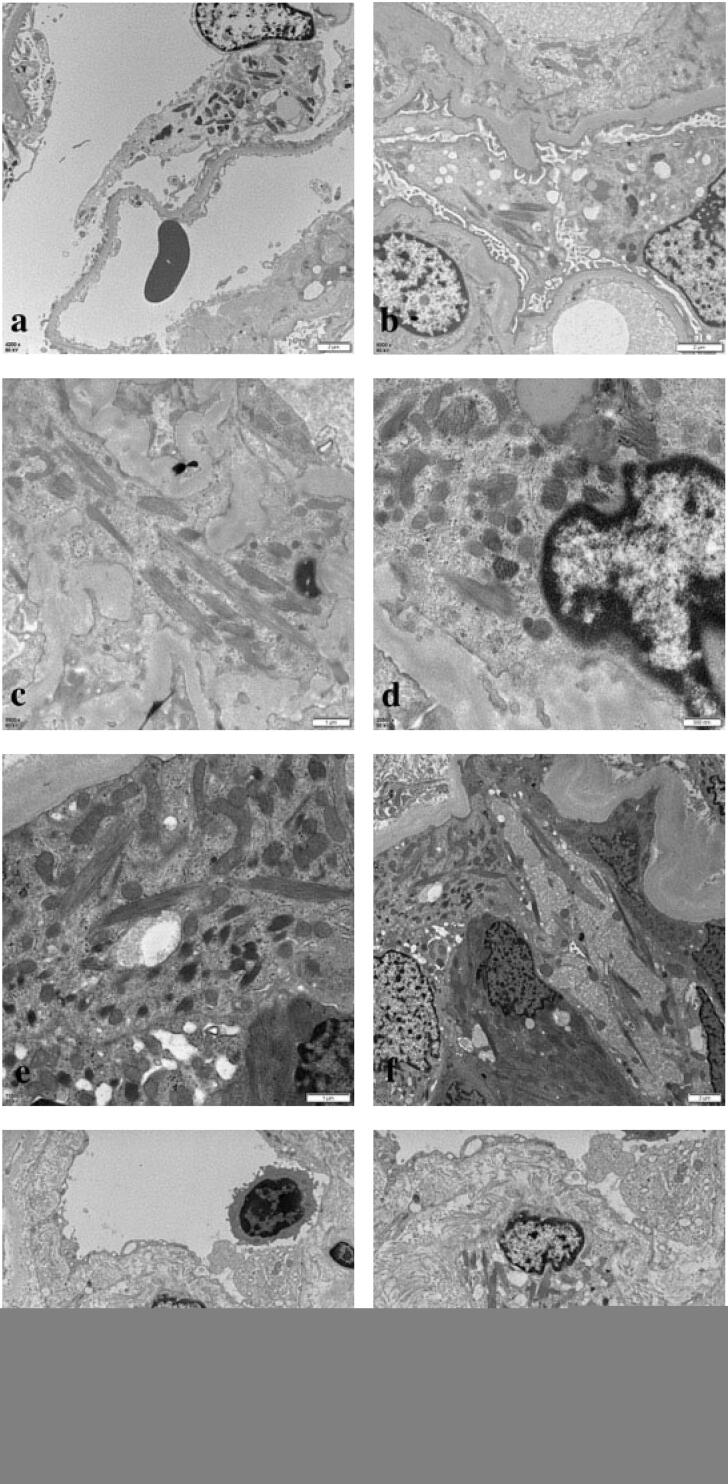
Electron microscopy showed crystalline inclusions in the podocytes (a, b), mesangial cells (c, d), proximal tubular epithelial cells (e, f), and histiocytes (g, h) (uranyl acetate and lead citrate staining, original magnification: a, f, g: ×4, 200, bar = 2 μm; b: ×6, 000, bar = 2 μm; c: ×9, 900, bar = 1 μm; d: ×20, 500, bar = 500 nm; e: ×11, 500, bar = 1 μm; h: ×6, 000, bar = 2 μm).

**Figure 8.**
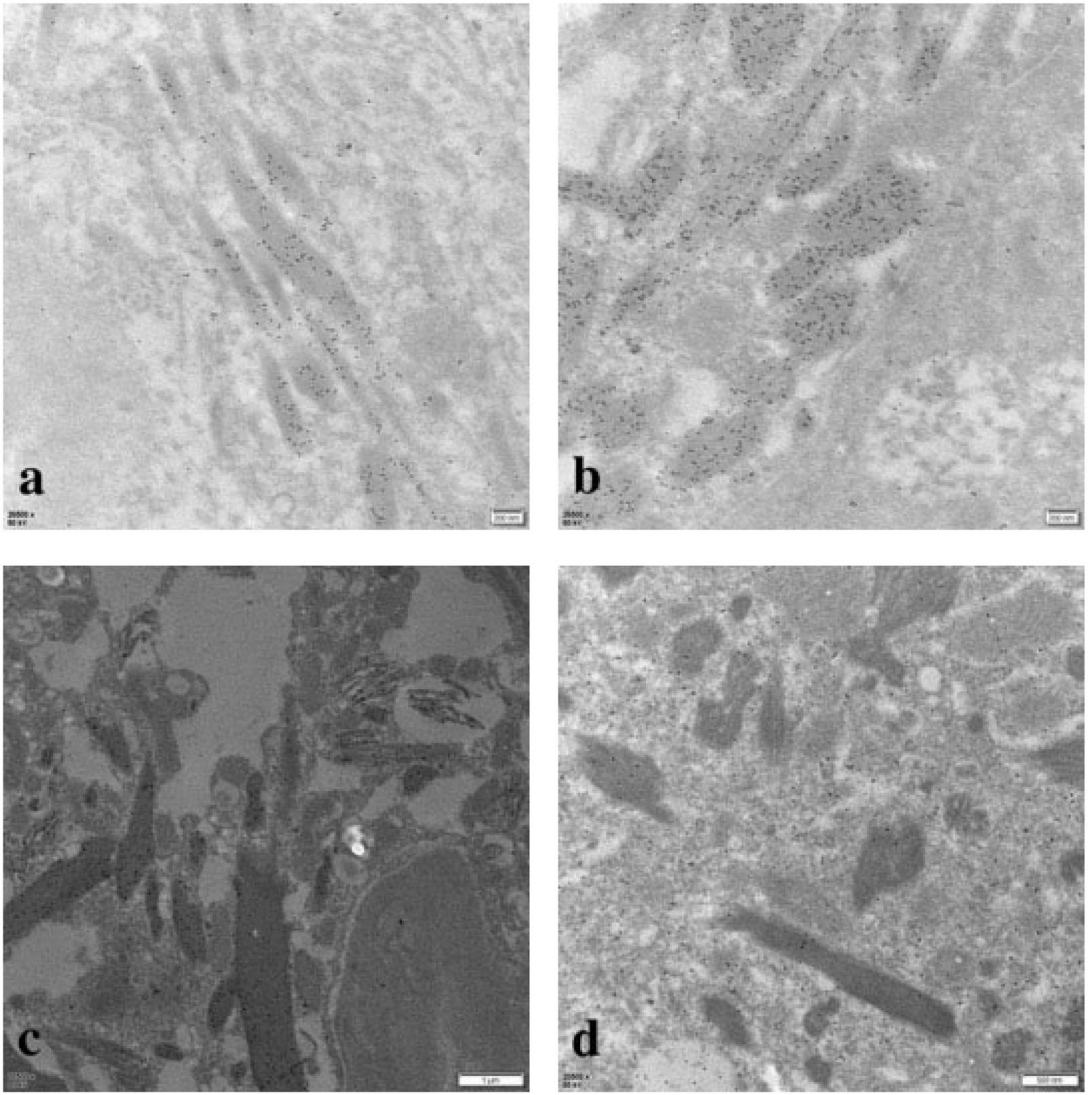
Immunoelectron microscopy showed that kappa light chain was strongly positive (a, b, c) in the crystalline inclusions and lysosomes of proximal tubular epithelial cells and podocytes, while lambda light chain was negative (d) (original magnification: a, b: κ × 26, 500, bar = 200 nm; c: κ × 11, 500, bar = 1 μm; d: λ × 20, 500, bar = 500 nm).

Renal biopsy revealed a total of 31 glomeruli, of which nine were globally sclerosed. Proximal tubular lesions were seen with focal atrophy and brush border disappearance. Eosinophilic needle-shaped crystals in the cytoplasm of the proximal tubular epithelial cells and podocytes were also observed. The same crystals were also noted in tubular cells shedded into tubular lumens and in some interstitial histiocytes and mesangial cells (Figure 2). Proximal tubular lumen was engorged by abundant small periodic acid–Schiff (PAS)-positive casts, which were granular rather than regularly fractured and homogeneous as seen in CN (Figure 3). The renal interstitium exhibited interstitial fibrosis. Diffuse lymphocytes, mononuclear cells, and histiocytes could be seen in the renal interstitium, without eosinophils. Congo red staining for amyloid deposition was negative. On immunohistochemistry, the histiocytes were positive for CD68 and kappa, and were negative for lambda (Figures 4 and 5). Direct immunofluorescence on frozen tissue was initially negative, but ultimately kappa positivity was confirmed with pronase digestion on formalin-fixed paraffin-embedded tissue (Figure 6). Electron microscopy showed irregular effacement of the podocyte foot processes. The number of lysosomes in the cytoplasm of the proximal tubular epithelial cells was significantly increased, and rods and needlelike crystalline materials composed of 20–40 nm fibrous materials were arranged in parallel and longitudinally. Granular and needle-shaped crystals could also be seen in the podocytes, mesangial cells, and histiocytes (Figure 7). To further distinguish the crystalline composition, immunoelectron microscopy was performed. This showed particulate kappa staining of the crystals and no staining for lambda (Figure 8). Kappa light chain-restricted CSH, LCPT, and LCCP was diagnosed. Although crystals also presented in the mesangial cells, no existing nomenclature was able to describe it.

A final diagnosis was assigned as MM with CSH involving both the bone marrow and kidney, with complications of LCPT and LCCP.

## Treatment and outcome

The patient was treated using induction chemotherapy with a combination of dexamethasone and bortezomib (BD) for two cycles. Serum creatinine levels decreased to 3.39 mg/dL, and urine output was normal. The patient then withdrew from dialysis and stopped chemotherapy. Although no specific treatment was introduced, the patient remained in a persistent clinically and biologically stable condition within a 2.5-year follow-up period, with serum creatinine of 3.39–3.85 mg/dL (normal, 0.51–0.95 mg/dL), hemoglobin of 79–94 g/L (normal, 115–150 g/L), platelet of 66–100 × 10^9^/L (normal, 125–350 × 10^9^/L). Osteodynia (mainly in the ribs) still presented intermittently, but the patient remained satisfied overall with her quality of life.

## Literature review

Renal involvement in CSH is poorly documented. We performed a systematic PubMed search with the term ‘crystal-storing histiocytosis’, and identified 155 relevant articles from 1987 to 15 October 2021. We identified 158 cases of CSH including ours. Twenty-nine of the cases included renal involvement and the following information was collected: age, sex, serum creatinine, urine protein, clinical manifestation, type of monoclonal protein, hematologic disease, distribution of crystals, extrarenal involvement, and outcomes (Table 1).

**Table 1.** Overview of CSH with renal involvement.

	Author [Ref]	Age/Sex	Scr (mg/dL)	Urine protein	Renal clinical manifestations	Monoclonal protein	Hematologic disease	Distribution of crystals			Cast nephropathy	Extrarenal involvement	Outcome
								Glomerulus	TE	Histiocyte			
1	Gupta et al. [4]	71/M	1.18	3.6	CKD	IgMκ	LPL	G-Cl	Yes	Yes	No	None	N/A
2	Gupta et al. [4]	67/M	2.7	Yes	AKI	IgGκ	MGRS	G-Cl	No	Yes	No	None	Died of MODS
3	Gupta et al. [4]	74/M	3.5	2	AKI	κ	MM	G-Cl	No	Yes	No	None	N/A
4	Galeano-Valle et al. [5]	65/M	7.6	N/A	AKI	κ	MGRS	No	lumen	No	No	BM, liver, spleen, LN	Died of GI hemorrhage
5	Wu et al. [6]	48/M	12.6	N/A	CKD	κ	MM	No	Yes	Yes	No	None	Stable, CTX, HD
6	El et al. [7]	52/M	2.9	0.29	CRF + FS	IgMκ	MGRS	N/A	N/A	N/A	No	BM	Died of sepsis, 25 years
7	El et al. [7]	70/M	2.4	0.8	CRF + FS	IgMκ	DLBCL	N/A	N/A	N/A	No	None	Died of sepsis, 14 months, Scr 3.2 mg/dL
8	El et al. [7]	65/M	6.3	2.5	CRF + FS	IgGκ	MGRS	N/A	N/A	N/A	No	None	Stable, 19 months, HD
9	Duquesne et al. [8]	62/F	normal	3.2	FS	IgGκ	MGRS	No	Yes	Yes	No	BM, eye	Stable, CTX, 18 months, CR
10	Ungari et al. [9]	66/F	1.6	1.95	CKD	κ	MM	No	Yes	Yes	No	BM	Stable, CTX, 6 months
11	Sethi et al. [10]	58/M	3.3	0.51	AKI	IgMλ	MZL	mes	No	Yes	No	None	Stable, CTX
12	Pitman et al. [11]	70/F	2.8	NA	AKI	κ	MGRS	No	No	Yes	No	None	Died of stroke, 6 months
13	Farooq et al. [12]	66/M	2.5	NA	AKI	IgGκ	MM	No	Yes	Yes	No	BM	Stable, CTX-ASCT, 6 months
14	Aline-Fardin et al. [13]	69/M	1.0	0.69	No FS	κ	MGRS	No	Yes	Yes	No	BM, LN	Stable, CTX, 12 months, CR
15	Keane et al. [14]	65/M	abnormal	NA	CKD	κ	MGRS	Yes	Yes	Yes	No	BM	N/A
16	Stokes et al. [15]	41/M	4.2	20.2	CKD + FS	IgDκ	MM	No	Yes	Yes	Yes	None	Stable, CTX-ASCT, 6 months, CR, Scr 2.2 mg/dL
17	Weichman et al. [16]	45/M	eGFR 64	0.7	None	IgGκ	MM	No	Yes	Yes	No	None	Stable, CTX-ASCT, CR
18	Tholouli et al. [17]	62/F	N/A	N/A	AKI	IgGκ	MM	No	Yes	Yes	No	None	Stable, CTX, 6 months, CR
19	Takahashi et al. [18]	60/M	N/A	N/A	CKD	IgAκ	MM	No	No	Yes	No	BM, liver, spleen	Died of electrolyte imbalance, 3.75 years
20	Kapadia et al. [19]	78/F	N/A	N/A	AKI	IgMκ	LPL	N/A	N/A	N/A	No	head and neck, LN	Died of AKI, CTX, 5 months
21	Garc-ia et al. [20]	44/M	N/A	N/A	N/A	IgGκ	LPL	No	No	Yes	No	BM, GI-Tract, Liver, heart, lung, spleen, head and neck, LN, pancreas, pleura and mesentery	Died of MODS, CTX, more than 1 year
22	Dogan et al. [21]	60/M	7	6.02	CKD	IgGκ	MM	VEC, mes	Yes	Yes	No	BM, eye	Stable, CTX, HD
23	Reeders et al. [22]	57/M	2.0	NA	CKD	IgGκ	LPL	VEC, endo, mes	Yes	Yes	No	Retroperitoneal area	N/A
24	Matthai et al. [23]	48/F	6.42	4.6	AKI + NS	κ	MM	VEC	Yes	Yes	Yes	None	N/A
25	Papla et al. [24]	51/M	abnormal	NA	CKD	κ	MM	VEC, endo, mes	Yes	Yes	No	BM, liver, lung	Died of MODS, 1 year
26	Nakamura et al. [25]	66/F	4.72	2.2	AKI	κ+ B-J	MGRS	VEC	Yes	Yes	Yes	None	N/A
27	Yamamoto et al. [26]	75/M	7.13	N/A	N/A	IgGκ	MM	VEC	Yes	Yes	No	BM, eye	Died of septic, CTX, 5 year
28	Ito et al. [27]	65/F	1.94	2.0g/gCr	AKI	IgGκ	MGRS	endo	Yes	Yes	No	None	Stable, CTX, 9 courses
29	Present	69/F	11.1	2.66	AKI on CKD	IgGκ + κ	MM	VEC, mes	Yes	Yes	No	BM	Stable, CTX, 3 years

Abbreviations: AKI: Acute kidney injury; ASCT: Autologous stem cell transplant; BM: Bone marrow; CKD: Chronic kidney disease; CTX: Chemotherapy; DLBCL: Diffuse large B-cell lymphoma; endo: Glomerular endothelial cell; F: Female; FS: Fanconi syndrome; G-Cl: Glomerular capillary loops; GI: Gastrointestinal; HD: Hemodialysis; LN: Lymph node; LPL: Lymphoplasmacytic lymphoma; M: Male; mes: Mesangial cell; MGRS: Monoclonal gammopathy of renal significance; MM: Multiple myeloma; MODS: Multiple organ dysfunction syndrome; MZL: Marginal zone lymphoma; N/A: Not available; Scr: Serum creatinine; TE: tubular epithelial cell; VEC: Visceral epithelial cell.

Renal involvement was recorded 18.4% of CSH cases; 46.8% of which were localized CSH, with the remainder being generalized CSH. Thirteen cases had bone marrow involvement, which was the most common organ involved in renal CSH. The average age at diagnosis was 62 years (range, 41–78 years), and the condition is more common in males (20 men and 9 women).

MM was the most common hematologic disease of renal CSH (13/29 cases), and IgGkappa was the most common type of monoclonal protein involved (12/29 cases). Kappa light chain was the most common light chain in found in renal CSH (28/29 cases). Needle-shaped crystals were found most commonly in the histiocyte cells in the renal interstitium, and in the perirenal fat. In some cases, the condition coexisted with LCPT (18/25), LCCP (8/24), LCCN (3/29), and crystalline deposition in the mesangial cells (4/24).

Only eight cases of combined CSH, LCPT, and LCCP were reported (the final eight cases in Table 1, including four cases with crystals in the mesangial cells). These tended to present with massive proteinuria, renal insufficiency, and Fanconi syndrome, while generalized CSH was more common. The prognosis among these patients appear to be seems better than in classic MM patients.

## Discussion

We describe a rare case of MM with multi-organ-involved CSH, coexisting with LCPT, LCCP, and crystals in the mesangial cells. Because of the rarity of this condition, few cases have been reported, and there is a lack of recognition of this disease. The diagnosis can be therefore very challenging. Here, we propose a new nomenclature of monoclonal immunoglobulin-induced crystalline nephrology to describe the coexistence of renal CSH, LCPT, LCCP, and crystalline deposition in the mesangial cells.

Monoclonal immunoglobulin inclusions or crystalline deposition are a rare type of monoclonal immunoglobulin-related disease, which usually includes LCPT, CSH and crystalglobulin glomerulonephritis. LCPT and CSH often coexist, suggesting that they may have the same pathogenesis [28]. Both conditions are so rare that they are often misdiagnosed.

The mechanism of crystalline formation in CSH is still unclear, but it may be related to unique mutations in the VK1 subgroup of the monoclonal kappa light chain, which can resist lysosomal proteolytic hydrolysis and promote crystal deposition by replacing polar residues with hydrophobic residues [7,29]. This is similar to the pathogenesis of LCPT [30,31]. In addition, patients may have inherited or acquired histiocytes processing defects that lead to crystal formation [32]. Excessive endocytosis of light chain can also induce oxidative stress in the proximal tubular cells, which directly activate inflammatory pathways through cytokines and Toll-like receptors, leading to inflammatory responses and LCPT; thereby damaging renal function [33].

The renal pathology of CSH usually presents with the deposition of needle-shaped crystals in the histiocyte cells of the renal interstitium (most common) and perirenal fat. They may also appear in the glomerular capillary loop and mesangial area, and often coexist with LCPT, LCCP, and/or CN. CD68 staining helps to determine the phenotype of histocytes, while electron microscopy is helpful for its diagnosis [4,34,35]. Immunoelectron microscopy helped us to identify the crystal composition. The pathology of LCPT is characterized by the presence of needle-shaped or granular hypereosinophilic and PAS-negative crystals within proximal tubular cells [34]. Some of them are along with LCCP. LCCP is extremely rare and must coexist with LCPT [29,35]. Our case demonstrates that immunoelectron microscopy is much more sensitive and can be highly effective in this setting, even though it is not routinely used. We propose a new nomenclature of monoclonal immunoglobulin-induced crystalline nephrology to describe the coexistence of renal CSH, LCPT, LCCP, and the crystalline deposition in mesangial cells, as their underlying mechanisms are similar.

The most common renal impairment in MM patients is CN, which mostly presents when the serum free light chain is higher than 100 mg/dL, and rarely occurs in patients with levels less than 70 mg/dL [6]. In our case, the serum free light chain was significantly increased, but no CN occurred, which may indicate that the structure and characteristics of the light chain are more decisive than their quantity. CN can also present in some MM patients with CSH combined with LCPT, as reported previously [6,34]. In our case, CSH, LCPT, and LCCP were coexistent together with special casts, rather than CN. The casts in our case were granular, heterogeneous, and PAS-positive, while the casts in CN are PAS-negative and homogeneous. It was demonstrated that these special casts came from the crystals in the necrotic proximal tubular epithelial cells. The overall process changes ranged from swelling of the cell to focal tubular epithelial necrosis and apoptosis with desquamation of cells into the tubular lumens. It should be recognized how easily this may be misdiagnosed as CN.

The clinical manifestation of CN is usually rapid progressive acute kidney injury. Our patient presented with acute kidney injury which progressed more slowly than in CN. The pathogenesis of CN involved casts composed of the light chains from overflow proteinuria which obstructed the distal convoluted tubules and collecting ducts [36]. The impairment of casts in our case seemed non-synchronized because of the coexistence of lesions in different periods, such that the acute kidney injury fluctuated and progressed relatively slowly. There was a rapid decrease in the level of serum creatinine without specific treatment on admission, which demonstrated the presence of a recovering acute kidney injury. This should be attributed to acute tubular necrosis, which was associated with the special casts. These pathological findings are consistent with the clinical manifestations.

Our patient presented with renal insufficiency and renal glycosuria. Although serum creatinine was significantly elevated, uric acid and serum phosphorus were normal, and hypokalemia persisted, which suggests the presence of Fanconi syndrome. Urinary protein was mainly low molecular weight protein, and albumin was also present. It was considered that the lesions mainly involved the proximal renal tubules and glomerulus. Due to the presence of monoclonal immunoglobulin, monoclonal gammopathy of renal significance (MGRS) or MM was suspected. Renal biopsy and bone puncture confirmed our hypothesis. It is suggested that monoclonal immunoglobulin-induced crystalline nephrology should be confirmed when Fanconi syndrome is complicated with monoclonal immunoglobulin. If there is evidence of glomerular involvement at the same time, caution should be taken with LCCP.

The best treatment for monoclonal immunoglobulin-induced crystalline nephrology is currently unclear, and chemotherapy remains the main treatment [37]. Consistent with our patient, MM patients with renal pathology of LCPT and/or CSH progress slowly and generally have a better prognosis than those with extracellular light chain deposition (CN or crystalglobulin glomerulonephritis), and also have a longer median survival than other MM patients. The reason may be that CSH patients are often diagnosed in the early stages of MM [5,38].

We found that in the diagnosis of crystals deposition, false negatives for light chains might sometimes present due to their intracellular localization, extensive crystallization, and the difficulty in exposing antigenic epitopes of the tertiary structure of light chain crystals in frozen tissue by standard immunofluorescence methods. When light chain crystal deposition is suspected, paraffin-embedded tissue should be digested with protease for antigenic repair and conformation change to better expose tissue antigenic clusters, to improve sensitivity and avoid missed diagnosis. This is consistent with the literature review [9,29,34].

Renal CSH combined with LCPT and LCCP, which we prefer to define as monoclonal immunoglobulin-induced crystalline nephrology, is extremely rare. Our patient was finally successfully diagnosed by multiple pathological techniques, such as paraffin-embedded tissue after pronase digestion, immunohistochemistry, and immunoelectron microscopy. Some limitations remain. Due to patient refusal, positron emission tomography–computed tomography was not obtained, which could have further confirmed whether other organs were involved. Although bone marrow aspirate sample and flow cytometry immunophenotypic analysis proved that the intracellular crystalline was kappa light chain-restricted, further immunohistochemical tests were absent. Due to patient refusal, the patient did not receive timely chemotherapy; however, her disease progressed very slowly during the follow-up period, which demonstrated the better prognosis of MM combined with monoclonal immunoglobulin-induced-crystalline nephrology.

## Conclusions

We report a rare case of combined generalized CSH, LCPT, and LCCP in a patient with MM, and propose a new nomenclature of monoclonal immunoglobulin-induced crystalline nephrology. In the presence of monoclonal immunoglobulin and Fanconi syndrome, we should be aware of the possibility of CSH and LCPT. Histological examination is crucial for the diagnosis of CSH, which may be underreported due to the difficulty of diagnosis. We proved that paraffin-embedded tissue after pronase digestion is beneficial to improve the sensitivity of diagnosis. Immunoelectron microscopy helps to determine the composition of the crystals. Through this literature review, we summarize the characteristics of renal CSH to improve recognition and diagnostic accuracy of this condition.

## Data Availability

All data generated or analyzed during this study are included in this published article.
